# Simple Estimation of Förster Resonance Energy Transfer (FRET) Orientation Factor Distribution in Membranes

**DOI:** 10.3390/ijms131115252

**Published:** 2012-11-19

**Authors:** Luís M. S. Loura

**Affiliations:** 1 Faculty of Pharmacy, University of Coimbra, Health Sciences Campus, Azinhaga de Santa Comba, 3000-548 Coimbra, Portugal; E-Mail: lloura@ff.uc.pt; Tel.: +351-239-488-485; Fax: +351-239-827-126; 2 Centre for Chemistry-Coimbra, Rua Larga, 3004-535 Coimbra, Portugal

**Keywords:** fluorescence, FRET, kappa-squared, linear dichroism, lipid bilayer, membrane probe, molecular dynamics

## Abstract

Because of its acute sensitivity to distance in the nanometer scale, Förster resonance energy transfer (FRET) has found a large variety of applications in many fields of chemistry, physics, and biology. One important issue regarding the correct usage of FRET is its dependence on the donor-acceptor relative orientation, expressed as the orientation factor κ^2^. Different donor/acceptor conformations can lead to κ^2^ values in the 0 ≤ κ^2^ ≤ 4 range. Because the characteristic distance for FRET, *R*_0_, is proportional to (κ^2^)^1/6^, uncertainties in the orientation factor are reflected in the quality of information that can be retrieved from a FRET experiment. In most cases, the average value of κ^2^ corresponding to the dynamic isotropic limit (<κ^2^> = 2/3) is used for computation of *R*_0_ and hence donor-acceptor distances and acceptor concentrations. However, this can lead to significant error in unfavorable cases. This issue is more critical in membrane systems, because of their intrinsically anisotropic nature and their reduced fluidity in comparison to most common solvents. Here, a simple numerical simulation method for estimation of the probability density function of κ^2^ for membrane-embedded donor and acceptor fluorophores in the dynamic regime is presented. In the simplest form, the proposed procedure uses as input the most probable orientations of the donor and acceptor transition dipoles, obtained by experimental (including linear dichroism) or theoretical (such as molecular dynamics simulation) techniques. Optionally, information about the widths of the donor and/or acceptor angular distributions may be incorporated. The methodology is illustrated for special limiting cases and common membrane FRET pairs.

## 1. Introduction

Fluorescence spectroscopy has been utilized as a major tool in biophysics in general, and membrane studies in particular, for decades. Several parameters can be measured in steady-state (quantum yield, anisotropy) or time-resolved (intensity and anisotropy decays) conditions, and it allows monitoring of photophysical processes that are sensitive to distance, concentration (Förster Resonance Energy Transfer, FRET), aggregation (FRET, static quenching) or diffusion (collisional quenching). Fluorescence’s versatility and sensitivity have earned it a place among the most useful biophysical techniques [1]. In particular, FRET is a most useful approach to the investigation of diverse problems in membrane biophysics, including membrane protein mapping, lateral heterogeneity (membrane domains), determination of the transverse location (depth) of fluorescent residues/labels inside the membrane, protein/lipid selectivity (preference of a specific lipid for the protein vicinity), and membrane protein oligomerization [2], both in spectroscopic studies and, more recently, under the microscope [3].

Additionally, quantitative applications of membrane fluorescence studies to the recovery of structural and partition information frequently require, as input, results obtained from other techniques. Some of these can be measured experimentally, such as area/lipid, lipid molar volume or bilayer thickness. However, one parameter for which there is no experimental technique suited to a definite measurement (though it was shown by Dale and coworkers that intervals containing it can be inferred from adequate fluorescence anisotropy measurents [4]) is the FRET orientation factor, κ^2^. Because the characteristic distance for FRET, *R*_0_, is proportional to (κ^2^)^1/6^, uncertainties in the orientation factor are reflected on the quality of information that can be retrieved from a FRET experiment. Most often, the theoretical value for the so-called dynamic isotropic limit (<κ^2^> = 2/3) is used, but <κ^2^> uncertainty is still widely regarded as an inconvenience [5] that may be especially important in membranes, because of their intrinsic anisotropic nature and the restricted rotational mobility experienced by fluorophores incorporated inside the bilayer. The 2/3 value corresponds to the dynamic limit, where rotation of both donor and acceptor is fast compared with the excited state lifetime.

As described in detail below in Section 3.1., the instant value of κ^2^ for a given donor-acceptor pair (which falls in the 0 ≤ κ^2^ ≤ 4 range) can be calculated from adequate molecular frame vectors. Although this definition of κ^2^ is not suited to experimental measurement, it can be conveniently used in molecular simulation studies. In particular, from the instant position coordinates of a molecular dynamics (MD) simulation trajectory, calculation of the orientation factor for a given FRET donor-acceptor molecular pair is straightforward, and averaging both over pairs and over time is conveniently carried out. This has been used in a number of literature reports (e.g., [6–9]) including membrane-embedded fluorophores [10]. However, this approach is not a choice for most experimental researchers, as setup and validation of MD simulations for novel fluorophores (for which parameterization is seldom available) is not trivial.

Even though computational calculations of κ^2^ are scarce, determination of membrane-embedded fluorophore orientations using computational (for reviews of MD studies of fluorescent membrane probes, see [11,12]) or experimental techniques (traditionally linear dichroism or other spectroscopic methods [13,14], or, more recently, methods based on polarized total internal reflection fluorescence microscopy or other microscopic techniques [15–17]) are more frequent.

This manuscript describes a simple manner to use data on probe location and orientation or alignment in membranes, obtained by experimental or molecular simulation methods, to provide estimates for the κ^2^ distribution in the dynamic limit, from numerical simulation. For this purpose, a simple program was developed to generate random planar location distributions of donor and acceptor probes. Fluorophore orientation is then generated for each donor or acceptor taking into account information provided by the user. Subsequently, κ^2^ is calculated for a large number of donor/acceptor pairs, and hence the κ^2^ distribution and average value are obtained. Average κ^2^ values for common membrane FRET pairs were obtained and are shown. For other FRET applications, the reader may use the software, which is available as supporting information to this article, without difficulty. This simplified approach enables immediate estimation of κ^2^ distributions for quantitative FRET applications, without the need to resort to sophisticated theoretical or experimental techniques.

## 2. Results and Discussion

### 2.1. Special Theoretical Cases

This manuscript describes a simple manner to use data on probe orientation or alignment in membranes to estimate the distribution of the κ^2^ FRET factor, using a small program described in more detail in Section 3. In this subsection we use the program to verify some results known from the literature, as well as to investigate other hypothetical scenarios of interest.

#### 2.1.1. Isotropic Dipole Distribution

**Figure 1:**
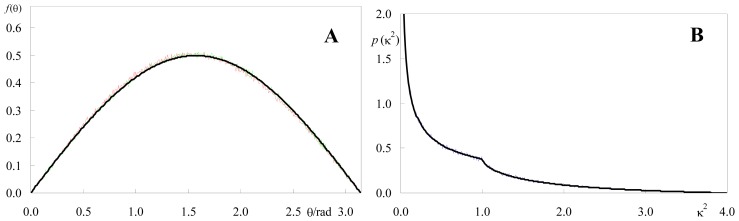
(**a**) Comparison of the simulated donor (red) and acceptor (green) density probabilities of orientation for planar distribution with the uniform distribution, sinθ/2 (black); (**b**) Comparison of the simulated κ^2^ density function (blue) with the theoretical curve of Equation 1 (black). <κ^2^> = 0.66. Inputs: coplanar distribution of donor and acceptor, θ_D,MAX_ = θ_A,MAX_ = 90°, σ_A_ = σ_B_ = 3; *N* = 10^7^ donor-acceptor pairs.

As described in Section 3, the software assumes normal distributions of cosθ_D_ and cosθ_A_ (truncated to the range −1 ≤ cosθ ≤ 1), where θ_D_ and θ_A_ are the tilts of donor and acceptor (respectively) relative to the normal to the membrane plane. The angles corresponding to the distribution maxima are read, as well as the standard deviations σ_A_ and σ_B_. If very wide angular distributions are used (σ >> 1) uniform azimuth angular distributions *f*(θ) = sinθ/2 are in practice obtained, as illustrated in Figure 1A. Therefore, and because ϕ distribution is uniform in the [0, 2π] interval, isotropic orientation of both donor and acceptor is easily simulated.

Figure 1B shows the resulting probability density of κ^2^ compared with the analytical result for isotropic dipoles, which is (e.g., [5]):

(1) $$p(\kappa^{2})=\{\begin{array}{ll} \frac{1}{2\sqrt{3\kappa^{2}}}\text{ln}(2+\sqrt{3}) & 0\leq \kappa^{2}\leq 1\\ \frac{1}{2\sqrt{3\kappa^{2}}}\text{ln}\left(\frac{2+\sqrt{3}}{\sqrt{\kappa^{2}}+\sqrt{\kappa^{2}-1}}\right) & 1\leq \kappa^{2}\leq 4 \end{array}$$

From the density distribution, one can calculate the average value,

(2) $$\langle \kappa^{2}\rangle =\int_{0}^{4}\kappa^{2}p(\kappa^{2})d\kappa^{2}=2/3$$

As shown in Figure 1B, there is excellent agreement between both simulated and analytical distributions and average κ^2^ value. The result <κ^2^> = 2/3, described in the literature [18] for planar probe distribution, is identical to that for isotropic orientation of dipoles distributed in a three dimensional system. The latter can also be obtained from the program, with a slight modification for the position of the acceptor in each pair (allowing a randomly allocated acceptor *z* value—varying e.g., between 0 and a maximal value specified by the user—in each donor-acceptor pair). Again, there is excellent agreement with the theoretical expectations (not shown).

#### 2.1.2. Identical or Identically-Distributed Fluorophores

**Figure 2:**
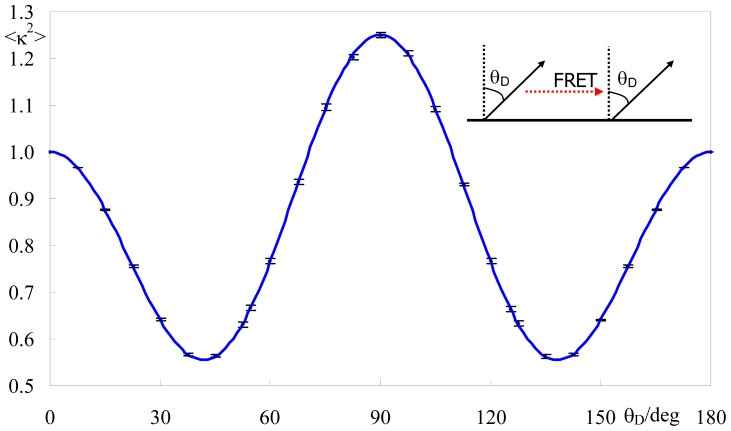
Average value of the orientation factor, <κ^2^>, as a function of the angle θ_D_, between the fluorophore transition and the normal dipole to the membrane plane, for two-dimensional FRET between donors and acceptors with identical orientation (see inset for illustration). Calculations with θ_D_ values spaced by 7.5° were carried out to obtain the points (the error bars length corresponds to two standard deviations of ten short simulations performed with *N* = 20,000 donor/acceptor pairs). The solid line is the analytical solution obtained by Knoester and van Himbergen [19].

This situation is illustrated in the inset of Figure 2. In this case, the distribution and average value of κ^2^ for planar systems depend solely on the angle θ_D_ between the donor transition dipole and the normal to the membrane plane. An analytical solution to this situation was described by Knoester and van Himbergen [19], who showed that

(3) $$\langle \kappa^{2}\rangle =\frac{2}{3}-\frac{2}{3}S+S^{2}$$

where

(4) $$S=\frac{3\langle {\text{cos}}^{2}\;\theta_{D}\rangle -1}{2}$$

is an orientation order parameter, equal to the average value of the second-order Legendre polynomial *P*_2_(θ). For this particular case, since no orientation heterogeneity is (as yet) assumed, <cosθ_D_> = cosθ_D_. This result, which arguably is little known in the membrane fluorescence research community, is however very pertinent e.g., for FRET among identical fluorophores (energy migration, energy transfer or homo-FRET).

**Figure 3:**
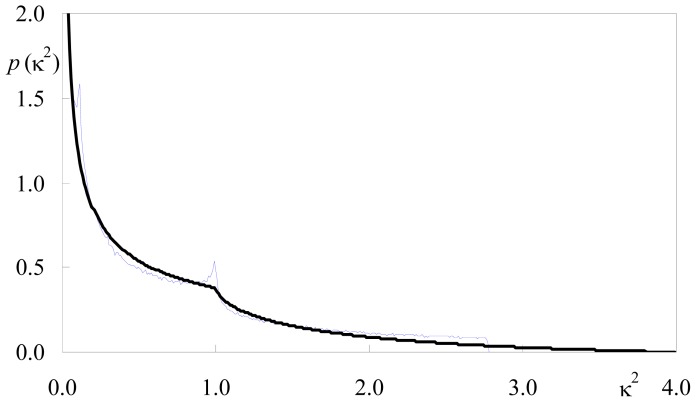
Comparison of the simulated κ^2^ density function for θ_D_ = θ_A_ = 54.74° (blue) with the isotropic theoretical curve of Equation 1 (black).

Figure 2 shows that the variation of <κ^2^> against θ_D_, obtained using our numerical approach, agrees with the analytical result. Large relative variations are observed, and between local maxima for θ_D_ = 0 (<κ^2^> = κ^2^ (invariant) = 1), 90° (<κ^2^> = 1.25) and 180° (<κ^2^> = κ^2^ (invariant) = 1), there are minima (<κ^2^> = 5/9) at θ_D_ = acos((5/9)^1/2^) = 41.81° and 180° – 41.81° = 138.19°. There are four θ_D_ values for which an average of 2/3 is obtained. These values correspond to the angles in the [0, 180°] interval for which *S* = 0 ↔ cosθ_D_ = ± (1/3)^1/2^ (the so-called magic angle 54.74°, and 125.26°) and *S* = 2/3 ↔ cosθ_D_ = ± (7/9)^1/2^ (28.13°, 151.87°). However, despite <κ^2^> being identical to the isotropic value, the κ^2^ distributions are quite different. As shown in Figure 3, the κ^2^ distribution for θ_D_ = 54.74° shows peaks at κ^2^ ≈ 0.12 and κ^2^ ≈ 1 (absent in the isotropic distribution), while it vanishes abruptly for κ^2^ > 25/9 = 2.778. This latter higher limiting value corresponds to the situation of coplanar donor dipole, acceptor dipole and separation vector, with the two dipoles facing each other. On the contrary, in the isotropic limit, values of κ^2^ near 4 (corresponding to collinear dipoles) are allowed and no cutoff is observed.

To illustrate the utility of this curve for quick estimation of <κ^2^>, we consider the recent MD study of 1-palmitoyl,2-[12-amino]dodecanoyl-*sn*-glycero-3-phosphocholine (C12-NBD-PC) in a fluid bilayer of 1,2-dipalmitoyl-*sn*-3-glycerophoshocholine (DPPC, *T* = 323 K). The angular distribution of the transition dipole has a maximum around ~120–125° relative to the bilayer normal [20]. From Figure 2, this would be consistent with <κ^2^> = 0.67–0.77. Direct calculation of κ^2^ using the atomic coordinates of the MD trajectory leads accordingly to <κ^2^> = 0.72 ± 0.04 for molecules in the same-leaflet [10].

### 2.2. Effect of Orientation Heterogeneity

One important feature of our numerical procedure for estimation of κ^2^ distributions of membrane-embedded fluorophores is that it allows the introduction of orientation heterogeneity, in the form of a normal distribution of cosθ_D_ and/or cosθ_A_, as mentioned previously. This type of heterogeneity is expected in simple rotational models such as the “wobbling-in-cone” motions [21]. The standard deviations of the cosθ_D_ and cosθ_A_ distributions (σ_D_ and σ_A_, respectively) are a measure of the extent of freedom of dipole orientation. Therefore, they serve a similar purpose to that of Dale *et al.*’s depolarization factors <*d*_D_^x^> and <*d*_A_^x^> [4]: σ and <*d*^x^> are different ways to express the orientation distributions sampled by donor and acceptor. Whereas Dale *et al.*’s form of taking this orientation redistribution into account using depolarization factors was justified by their perceived possibility of estimation of <κ^2^> ranges from time-resolved fluorescence polarization measurements, σ is a technique-independent, more direct metric of the dipole orientation heterogeneity.

**Figure 4:**
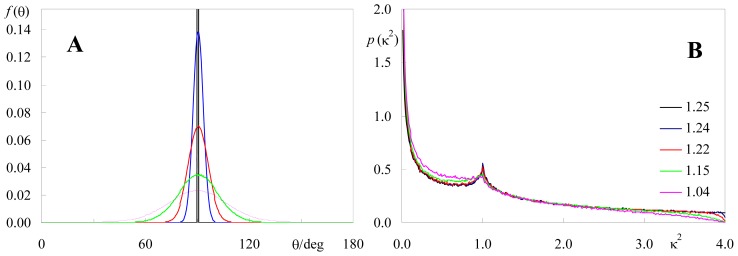
Effects of widening donor and acceptor dipole angular distributions (identical, centered around 90°, depicted in (**a**)) on the simulated κ^2^ density functions (**b**); Colors indicate standard deviations of cosθ distributions as follows: black, σ = 0; blue, σ = 0.05; red, σ = 0.10; green, σ = 0.20; magenta, σ = 0.30. The numbers given in the inset of (**b**) correspond to the average κ^2^ value. *N* = 10^6^ donor-acceptor pairs.

**Figure 5:**
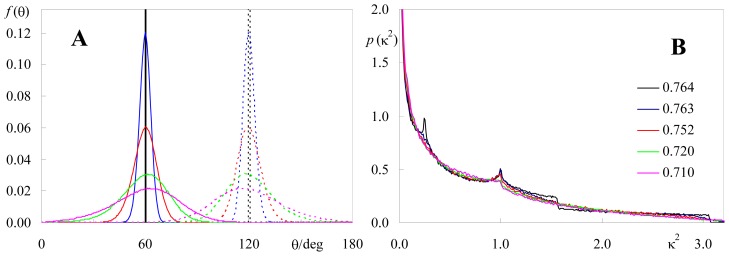
Effects of widening donor and acceptor dipole angular distributions (distinct, centered around 60° (donor, solid curves) and 120° (acceptor, dotted curves), depicted in (**a**)) on the simulated κ^2^ density functions (**b**); See Figure 4 for color scheme. The numbers given in the inset of (**b**) correspond to the average κ^2^ value. *N* = 10^6^ donor-acceptor pairs.

Intuitively, upon permitting sampling of different donor/acceptor relative orientations, κ^2^ distributions and average values are necessarily affected. As discussed above, the limiting case of σ >> 1 leads to the expected isotropic case. Here we address the more physical possibility of moderate heterogeneity (σ ≈ 0.05–0.3) and its effect upon the orientation factor. Figures 4 and 5 illustrate the alterations produced on κ^2^ distributions and average values upon widening the angular distributions of donor and acceptor dipoles for two distinct situations: identical donor and acceptor distributions (e.g., homo-FRET) centered around θ = 90° (Figure 4) and quite distinct donor and acceptor distributions centered around 60° and 120°, respectively (Figure 5). In these calculations, a larger number of pairs (at least 10^6^) should be considered, to allow a better sampling of the θ_D_ and θ_A_ distributions. In both cases, it can be seen that whereas moderate orientation heterogeneity produces visible alterations in κ^2^ distribution, significant effects require a very large degree of heterogeneity. This is more apparent for the system where donor and acceptor have very distinct angular distributions (Figure 5), where, remarkably, very wide distributions of both probes affect κ^2^ by a degree <10%.

### 2.3. Distance Effects

In most applications of intramolecular FRET (involving a single acceptor fluorophore as quencher of each donor), distance measurements are carried out assuming a single fixed value of *R*_0_, assuming a <κ^2^> value (usually 2/3) independent of donor-acceptor distance. However, this view has been challenged, as MD simulations revealed considerable correlation between κ^2^ and donor-acceptor distance in a labeled protein [6], possibly related to constraints imposed by the linker used to attach the donor probe. Naturally, in systems where each donor may transfer its excitation energy to multiple acceptors at varying distances, correlation between donor-acceptor distance and their relative orientation will necessarily occur if the dynamic isotropic limit is not met. Consider for example that donors and acceptors are located in separate parallel planes (different depths in the membrane), and that both types of fluorophores have narrow orientation distribution. The range and relative weight of possible κ^2^ values will certainly differ for a donor-acceptor pair in which the acceptor is located directly below or above the donor, relative to the case where their lateral separation is large. Therefore, the question is not whether there exists correlation, but to which extent κ^2^ may vary as a function of distance, and how to best take this into account.

**Figure 6:**
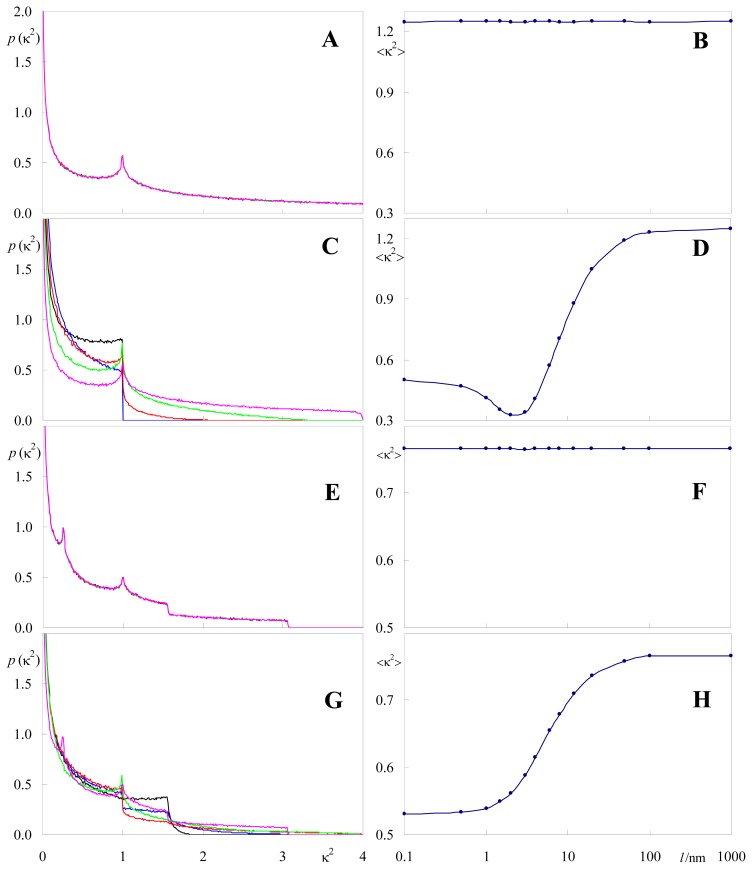
κ^2^ distributions (**a**,**c**,**e**,**g**) and average values (**b**,**d**,**f**,**h**) obtained for identical (centered around 90°; (**a**–**d**)) and distinct (centered around 60° (donor) and 120° (acceptor); (**g**–**h**)) fluorophore dipole distributions, both for coplanar donors and acceptors (**a**–**b**,**e**–**f**) and with parallel donor and acceptor planes (separated by *h* = 2.0 nm, (**c**–**d**, **g**–**h**)), for varying simulated system size, *l*. Colors in the left panels indicate the value of *l* (black, 1 nm; blue, 2 nm; red, 4 nm; green, 8 nm; magenta, 100 nm). All lines in panels (**a**) and (**e**) are essentially identical.

For the sake of illustration, we consider the same donor and acceptor orientations as in the previous subsection, but assuming fixed (no orientation heterogeneity) θ_D_ = θ_A_ = 90° and (θ_D_, θ_A_) = (60°, 120°). We also consider two separate possibilities in each case: (i) coplanar donor and acceptor spatial distributions; (ii) donor and acceptor planes separated by an arbitrary distance of *h* = 2.0 nm. In each of these scenarios, the size of the square systems *l* was varied up to 1000 nm. 10^6^ pairs were simulated for proper sampling of donor/acceptor distances. It is clear from the figure that location of both donor and acceptor dipoles in the same plane always lead to identical distributions (Figure 6A,E) and average values (Figure 6B,F). At variance, for donors and acceptors placed in parallel planes, there are very significant changes in both κ^2^ distribution (Figure 6C,G) and <κ^2^> (Figure 6D,H), when the size of the simulated system (which constraints the maximal possible donor-acceptor distance) is <<100 nm.

In this case, definition of an average κ^2^ becomes a problem. The best way to deal with this situation is to take notice of how FRET occurs from a given donor (with fluorescence lifetime in absence of acceptor equal to τ_0_) to a distribution of *M* acceptors (where the distance between the selected donor and the ith-acceptor is denoted by *R*_i_). The decay rate *k* is given by

(3) $$k=\tau_{0}^{-1}+k_{T}=\tau_{0}^{-1}+\sum_{i=1}^{M}k_{i}$$

where the different individual FRET rates are calculated according to

(4) $$k_{i}=\frac{1}{\tau_{0}}{\left(\frac{R_{0i}}{R_{i}}\right)}^{6}$$

Finally, the Förster radius for each donor-acceptor pair is calculated using

(5) $$R_{0i}=0.02108{\left[\kappa_{i}^{2}\Phi_{0}n^{-4}\int_{0}^{\infty }\lambda^{4}I(\lambda )ɛ(\lambda )d\lambda \right]}^{1/6}$$

where Φ_0_ is the quantum yield of the donor in the absence of acceptor molecules, *n* is the refractive index of the medium, and the integral term is the overlap between the normalized donor emission (*I*(*λ*)) and the acceptor absorption (*ɛ*(*λ*)) spectra. The constant value in the equation above assumes nm units for both λ and *R*_0_, as well as M^−1^cm^−1^ units for *ɛ*(*λ*). Combining these three equations, one concludes that the FRET rate is given by

(6) $$k_{T}=C\sum_{i=1}^{M}\frac{\kappa_{i}^{2}}{R_{i}^{6}}$$

where *C* is a constant term for all acceptors in the distribution. That is, the natural way to average κ^2^ over different acceptors, with different distances to a given donor, is to use the inverse sixth power of the donor-acceptor distance as weight. In this way, acceptors located more closely contribute significantly more to the average (as they are also responsible for most of the quenching by FRET). For the purpose of this calculation, we adapted our program for averaging κ^2^ in this way for FRET between a plane of donors and another of acceptors, and FRET in a bilayer geometry (with possibility of FRET between a plane of donors and two planes of acceptors—one in each bilayer leaflet). We then proceeded to calculate <κ^2^> for the two angular distributions presented above (θ_D_ = θ_A_ = 90°; and (θ_D_, θ_A_) = (60°, 120°)) assuming a bilayer geometry with *h*_1_ = 0.0 and *h*_2_ = 2.0 nm as the donor-acceptor interplanar distances. For coplanar fluorophores, an exclusion distance of *R*_e_ = 0.8 nm was also introduced, as donors and acceptors in the same plane cannot lie closer than a given minimum distance value. This would be similar to a typical experiment, with acceptor distribution in both bilayer leaflets. For a better sampling of all possible orientations for small *R*_i_ values, a value of *l* = 100 nm was used. Ten different simulations were carried out in each case, to assess whether proper convergence was obtained. For θ_D_ = θ_A_ = 90°, an average of 1.237 with standard deviation 0.010 was obtained, whereas for (θ_D_, θ_A_) = (60°, 120°) the corresponding values were 0.771 and 0.009. The relatively small standard deviations indicate that sampling was adequate. The average values were similar (very slightly lower) to the <κ^2^> calculated for the planar systems (1.250 and 0.776, respectively), and the small differences are due to the contribution of acceptors lying in the opposite leaflet. Because of the transverse distance of 2 nm, neglecting this leaflet does not lead to considerable error. In any case, for the following section that applies this methodology to computation of <κ^2^> for common experimental donor-acceptor pairs, whenever possible, information on donor-acceptor interplanar distances was taken into account.

### 2.4. Application to Experimental FRET Pairs

Table 1 summarizes relevant information regarding membrane probes that are commonly involved in FRET experiments (as donors, acceptors or both), Förster pair combinations formed by these probes, examples of quantitative experimental FRET studies using these pairs, and information obtained from literature simulation or experimental studies regarding fluorophore location and transition dipole orientation of the probes. The latter information was used to calculate an average κ^2^ value using the inverse sixth power of the donor-acceptor distance as weight, as described in the previous subsection. These averages are also shown in the Table. It can be seen that all these values but one (homo-FRET between Rhodamine B probes) lie within 15% or 0.1 of the isotropic limit of 2/3, even though the probe orientation distributions are quite varied. The most probable explanation for this is that all orientation data retrieved from the literature refer to liquid disordered bilayers, in which probes have generally broad angular distributions. Applications to FRET in liquid ordered or gel phases, where conformational freedom is largely reduced, would potentially lead to <κ^2^> further from the 2/3 value. This is illustrated in the *t*-PnA/DPH pair. For *t*-PnA, contrary to other probes, information regarding orientation distribution is also available in the gel phase (5°, 0.03 (gel) [22]). Using these input values instead of the fluid phase parameters, one would obtain <κ^2^> = 0.53 ± 0.01 instead of 0.58 ± 0.01 for both probes in the fluid phase. It should also be emphasized that the dipole orientation details shown in Table 1 refer to a specific probe compound labelled with the fluorophore under consideration, and other probes exist where the same fluorophore could be expected to show different transverse location and/or orientation distribution in the bilayer. For example, NBD data are based in a MD study of NBD-PC, whereas FRET studies also employ other NBD probes (NBD-PE, NBD-cholesterol).

### 2.5. Effect of <κ^2^> on FRET Efficiency

The most common experimental observables in FRET are the decay of donor in the presence of acceptor, *i*_DA_(*t*), and the FRET efficency, *E*. The decay law *i*_DA_(*t*) for bilayer geometries and uniform probe distribution is available in the literature [23]

(7) $$i_{DA}(t)=\text{exp}\left(-\frac{t}{\tau_{0}}\right)\text{exp}\left\{-\pi R_{0}^{2}c\gamma \left[\frac{2}{3},{\left(\frac{R_{0}}{R_{e}}\right)}^{6}\left(\frac{t}{\tau_{0}}\right)\right]{\left(\frac{t}{\tau_{0}}\right)}^{1/3}\right\}\cdot \text{exp}\left\{\pi R_{e}^{2}c\left(1-\text{exp}\left[-{\left(\frac{R_{0}}{R_{e}}\right)}^{6}\left(\frac{t}{\tau_{0}}\right)\right]\right)\right\}$$

In this equation, *c* is the number of acceptors per unit area, γ is the incomplete gamma function, and the other symbols have the same meaning as in the previous equations. Although Equation 7 was originally derived for a plane of acceptors containing the donor (*cis* transfer), it is also valid if the donor molecule is separated from the acceptor plane by a distance *R*_e_. From the time-resolved donor emission, the FRET efficiency, which is defined (and often experimentally measured) by

(8) $$E=1-I_{DA}/I_{D}$$

(where *I*_DA_ and *I*_D_ are the donor steady-state emission intensities in presence and absence of donor, respectively), can be calculated using

(9) $$E=1-\int_{0}^{\infty }i_{DA}(t)dt/\int_{0}^{\infty }i_{D}(t)dt$$

In the latter equation, *i*_D_(*t*) = exp(-*t*/*τ*_0_) is the donor time-resolved fluorescence decay in the absence of acceptor.

To appreciate the effect of <κ^2^> on the FRET efficiency, we first define $\bar{R_{0}}$ as the value of the Förster radius calculated with κ^2^ = 2/3, and then consider a form of Equation 7 in terms of dimensionless parameters ζ= *t*/*τ*_0_ (reduced time), $\sigma =\pi {\bar{R_{0}}}^{2}c$ (number of acceptors in a circle of radius equal to $\bar{R_{0}}$), $\alpha =\bar{R_{0}}/R_{\text{e}}$ and β = (3/2)<κ^2^>:

(10) $$i_{DA}(\varsigma )=\text{exp}(-\varsigma )\text{exp}\left\{-\beta^{1/3}\sigma \left(\gamma \left[\frac{2}{3},\beta \alpha^{6}\varsigma \right]\varsigma^{1/3}-\frac{1-\text{exp}\left(-\beta \alpha^{6}\varsigma \right)}{\beta^{1/3}\alpha^{2}}\right)\right\}$$

**Figure 7:**
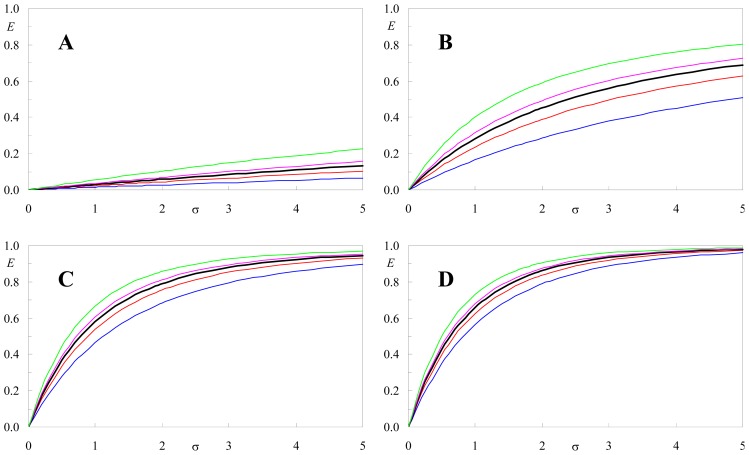
FRET efficiency *E* as a function of dimensionless acceptor concentration $\sigma =\pi {\bar{R_{0}}}^{2}c$, for given values of $\alpha =\bar{R_{0}}/R_{\text{e}}$ (panels ((**a**–**d**) correspond to α = 0.5, 1.0, 2.0, and 5.0, respectively) and <κ^2^> (in each panel, curves obtained with <κ^2^> = 0.30, 0.50, 2/3, 0.80 and 1.25 are show in blue, red, black, magenta and green, respectively).

This form of the decay law is suitable for calculation (by integration over ζ, done using Equation 9 after substitution of variables *t* and ζ) of universal curves *E vs.* σ, for fixed values of α and β. Representative examples are shown in Figure 7.

From Figure 7, it can be seen that variations in FRET efficiency upon changing the <κ^2^> value used to calculate *R*_0_ are most important when the latter is smaller than or comparable to the exclusion distance, such as in panels (**a**) and (**b**). For example, for $\alpha =\bar{R_{0}}/R_{\text{e}}=0.50$, the FRET efficiency obtained for <κ^2^> = 1.25 is >3 times larger than that obtained for for <κ^2^> = 0.30 over the whole σ < 5 range. However, it must be noted that larger values of $\alpha =\bar{R_{0}}/R_{\text{e}}$ are often met in membranes, as long as *R*_0_ has a reasonable (~2.5 nm or larger) value and there are acceptors in the same bilayer leaflet as the donors. In these cases, such as illustrated in panels (**c**) and (**d**), the effect of <κ^2^> on *E*, though still clearly detectable, is smaller, especially in the 0.50 < <κ^2^> < 0.80 range which is observed for most common FRET pairs (Table 1).

## 3. Methods

### 3.1. Definition of κ^2^ for a Given Donor-Acceptor Pair

**Figure 8:**
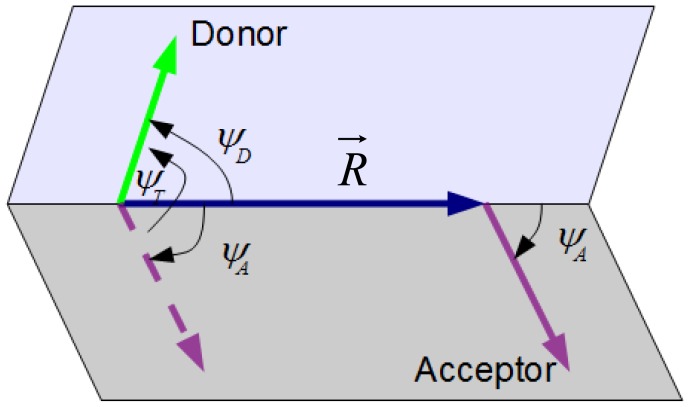
Vectors and angles relevant to the orientation factor. Adapted from [10].

κ^2^ is given by [5]

(11) $$\kappa^{2}={\left(\text{cos}\psi_{T}-3\;\text{cos}\psi_{D}\;\text{cos}\psi_{A}\right)}^{2}$$

where Ψ_T_ is the angle between the transition moments of the donor and acceptor and Ψ_D_ and Ψ_A_ are the angles between the donor and acceptor transition moments and the vector uniting their centers, *R⃗* (Figure 8). An equivalent expression, involving scalar products of &*rrarr;* = *R⃗*/||*R⃗*|| and unit vectors &*urarr;_D_* and &*urarr;_A_* (in the direction of the donor and acceptor dipoles, respectively), is

(12) $$\kappa^{2}={\left({\vec{u}}_{D}\cdot {\vec{u}}_{A}-3(\vec{r}\cdot {\vec{u}}_{D})(\vec{r}\cdot {\vec{u}}_{A})\right)}^{2}$$

### 3.2. Calculation of κ^2^ Distributions and Averages from Numerical Simulation

Two programs, written as macros in the Visual Basic Editor within a Microsoft Excel document, were developed for this article. The programs are available as supplemental files to this article, and may be edited by any user. Microsoft Excel Visual Basic was chosen as it may be easily used by most readers, and produces immediate numerical and graphic output.

Program 1 calculates κ^2^ distributions and average values without weighting, for a single acceptor plane. The basic algorithm can be summarized as follows:

1. Read inputs: θ_D,max_, θ_A,max_, σ_D_, σ_A_, *l*, *N*, donor-acceptor interplanar distance *h*, and the number of bins (equal length subintervals of 0 ≤ κ^2^ ≤ 4) for κ^2^ distribution.
2. Main cycle – calculate κ^2^ for the i^th^ donor-acceptor pair:
  2.1. Set donor and acceptor coordinates, (*x*_Di_, *y*_Di_, *z*_Di_) and (*x*_Ai_, *y*_Ai,_*z*_Ai_), respectively. For the *x* and *y*-coordinates, uniformly distributed random numbers between 0 and *L* are allotted. *z*_Di_ is taken as zero, whereas *z*_Ai_ is taken as *h*.
  2.2. Calculate donor-acceptor separating vector *R⃗*, and its modulus.
  2.3. Set donor and acceptor dipole orientation coordinates, (θ_Di_, ϕ_Di_) and (θ_Ai_, ϕ_Ai_), respectively. For ϕ_Di_ and ϕ_Ai_, uniformly distributed random numbers between 0 and 2π are allotted. θ_Di_ and θ_Ai_ (the angles relative to the bilayer normal, so that they fall between 0 and π) are calculated by firstly computing their cosines, using Gaussian random numbers centered around cosθ_D,max_ and cosθ_A,max_, with standard deviation σ_D_ and σ_A_, respectively. Gaussian random numbers are obtained using the Box-Muller transform method [51,52]. If either of the calculated cosines falls outside the [-1,1] interval, new cosθ_Di_ and cosθ_Ai_ are calculated. Otherwise, θ_Di_ and θ_Ai_ are obtained in the [0, π] interval by inversion.
  2.4. Calculate Cartesian coordinates of unit dipole vectors *u⃗_D_* and *u⃗_A_*, from (θ_Di_, ϕ_Di_) and (θ_Ai_, ϕ_Ai_).
  2.5. Calculate κ^2^ using Equation 12.
  2.6. Update histogram of κ^2^ distribution (in fact, the program also calculates θ_D_ and θ_A_ distributions, for testing and/or comparison and matching with distributions from the literature; these are also updated at this point), and partial sum of κ^2^ values (for calculation of <κ^2^> = sum(κ^2^_i_)/*N* at the end of the cycle).
  2.7 Increment i and repeat until i > *N*.
3. Write outputs to the active worksheet: κ^2^ average value and distribution, θ_D_ and θ_A_ distributions.

All calculations and distributions shown in Figures 1–6 were obtained with this program.

Program 2 follows a similar algorithm for calculation of κ^2^ for the i^th^ donor-acceptor pair. However, it computes an average κ^2^ value using *R*_i_^−6^ as weight for each pair. It may be used for both one and two acceptor planes. The program reads θ_D,max_, θ_A,max_, σ_D_, σ_A_, *l*, *N*, and, for both planes, the respective donor-acceptor interplanar (*h*_1_ and *h*_2_) and exclusion (*R*_e1_ and *R*_e2_) distances. Two main cycles are carried out, one for each acceptor plane, with calculation of κ^2^ distribution in each case. For both cycles, calculation of κ^2^ for the i^th^ donor-acceptor pair is identical to that in the first program, with an additional check: if *R*_i_ < *R*_e1_ (first cycle) or *R*_i_ < *R*_e2_ (second cycle), then new donor and acceptor distance coordinates are allotted and a corresponding new *R*_i_ is calculated. Partial sums of κ^2^/*R*^6^ values are now computed, to obtain <κ^2^> = sum(κ^2^_i_/*R*_i_^6^)/sum(1/*R*_i_^6^). As described in Section 3, this weighting scheme reflects the dependence of the FRET interaction with distance, and was used to compute the values of the right column of Table 1 (see footnote for typical input parameters).

## 4. Conclusions

A simple method for calculation of the distribution and average value of the FRET orientation factor κ^2^, for membrane-located donor and acceptor fluorophores, is presented. The method is implemented in two programs that are available with the present article. The programs use as inputs the donor and acceptor transverse locations, orientation and orientational heterogeneity. Analytical results previously obtained in limiting cases were verified, validating our approach. A number of donor/acceptor orientation distributions tested in this work produce κ^2^ distributions and average values which are significantly different to that corresponding to the isotropic limit, characterized by <κ^2^> = 2/3. Considerable dependence of <κ^2^> on system size *l* when the latter is <<100 nm was verified, which presents a problem regarding the most appropriate <κ^2^> to consider for the purpose of quantitative analysis of FRET data. It is proposed that calculating <κ^2^> using the sixth inverse power of the donor-acceptor distance is the most natural way to average κ^2^ in this situation, and estimates of <κ^2^> were obtained for common membrane FRET pairs using available fluorophore location and orientation data. It was shown that for most of these systems, the calculated <κ^2^> lies within ±15% of the 2/3 value, possibly because probes in fluid bilayers have very broad orientation distributions. In these cases, substantial effects on FRET efficiency are not expected, provided that the Förster radius is significantly larger than the donor-acceptor exclusion distance. For more ordered bilayers, narrow orientation distributions may produce <κ^2^> further form the 2/3 value. In these ordered systems, an additional problem is that fluorophore dipole orientation randomization may occur in a time scale of the order or slower than the donor excited state lifetime. In case reorientation proceeds much slower than transfer, the opposite regime (static limit) is operative. As discussed in detail elsewhere [4,5], in this limit, it is generally not possible to use κ^2^ averages to calculate donor-acceptor distances in intramolecular FRET (the only exceptions occur for isotropic dipole distribution or when the FRET efficiency is vanishingly small). In the intermediate case of comparable rates of reorientation and transfer, FRET kinetics becomes very complex [19]. Because these situations of restricted mobility are not contemplated in our method, which calculates κ^2^ distributions and averages corresponding to the dynamic limit, care must be taken when addressing these systems. However, even in such cases, the ease of application of this method—as long as fluorophore location and orientation data (or, at worst, “educated guesses”) are available (in the literature or upon measurement) for the pair at hand—allows calculation of <κ^2^> which will constitute an improvement over the widely used but very seldom justified <κ^2^> = 2/3 assumption.

## Figures and Tables

**Table 1:** Donor and acceptor location and orientation literature data for common FRET pairs in membranes, and <κ^2^> calculated from this information.

Donor fluorophore a	Acceptor fluorophore a	Quantitative experimental FRET studies	Donor transverse location b	Donor orientation (θ_max_, σ) c	Acceptor transverse location	Acceptor orientation (θ_max_, σ) c	Calculated <κ^2^> d
*t*-PnA	DPH	[24,25]	0.92 nm [26]^e^	20°, 0.15 f	0.75 nm [27]^f^	0, 0.35 [27]^f^	0.58 ± 0.01
DPH	NBD	[28–31]	0.75 nm [27]^f^	0, 0.35 [27]^f^	1.35 nm [20,32,33]^e^,^f^	125°, 0.35 [20]^f^	0.66 ± 0.01
NBD	NBD	[34,35]	1.35 nm [20,32,33]^f^	125°, 0.35 [20]^f^	1.35 nm [20,32,33]^e^,^f^		0.66 ± 0.03
NBD	Carbocyanine	[36]			1.26 nm [37]^f^	77°, 0.25 [37]^f^	0.76 ± 0.02
NBD	Rhodamine B	[25,35,38–42]			2.1 nm [43,44]^f^	90°, 0.275 [43,45]^g^	0.56 ± 0.02
BODIPY	Rhodamine B	[30,46]	0.8 nm [47]^f^	10°, 0.5 [47]^f^			0.57 ± 0.01
BODIPY	Carbocyanine	[48]			1.26 nm [37]^f^	77°, 0.25 [37]^f^	0.72 ± 0.02
Rhodamine B	Rhodamine B	[25,35,41,49,50]	2.1 nm [43,44]^f^	90°, 0.275 [43,45]^g^	2.1 nm [43,44]^f^	90°, 0.275 [43,45]^g^	1.08 ± 0.01

a *t*-PnA: *trans*-parinaric acid; DPH: diphenylhexatriene; NBD: 7-nitrobenz-2-oxa-1,3-diazol-4-yl)amino; BODIPY: 4,4-difluoro-3a,4adiaza-s-indacene;

b Relative to the center of the bilayer;

c The values given refer to the θ_max_ and σ values (read by the simulation program) that produce angular distributions most consistent with the simulation or experimental data reported in the cited references;

d Average and standard deviations of average for ten independent simulations with two planes, *R*_i_^−6^ weight, *N* = 10^6^, *R*_e_ = 0.8 nm and *l* = 100 nm;

e Inferred from experimental fluorescence quenching data;

f Inferred from simulation data;

g Inferred from linear dichroism data.
